# Advanced monitoring of harmful substances and their effects in the Baltic Sea is desired: A comment on Kanwischer et al. (2021)

**DOI:** 10.1007/s13280-022-01717-z

**Published:** 2022-03-15

**Authors:** Harri T. Kankaanpää, Raisa Turja, Kari K. Lehtonen

**Affiliations:** Marine Research Centre, Finnish Environment Institute, Latokartanonkaari 11, 00790 Helsinki, Finland

Comment to: Kanwischer, M., N. Asker, A.S. Wernersson, M.A. Wirth, K. Fisch, E. Dahlgren, H. Osterholz, F. Habedank, et al. 2021. Substances of emerging concern in Baltic Sea water: Review on methodological advances for the environmental assessment and proposal for future monitoring. *Ambio*. 10.1007/s13280-021-01627-6

In their review paper, Kanwischer et al. (2021) discuss contaminants of emerging concern (CEC), and the use of state-of-the-art analytical chemistry and effect-based methods (EBM) that currently receive too little attention in Baltic Sea monitoring programs and suggest new approaches. We provide additional information on this important topic.

The current review states that “current Baltic Sea monitoring programs do not address compounds of emerging concern.” Although not yet mandatory in MSFD, WFD or HELCOM context, they are addressed in many national monitoring programs. For example, pharmaceuticals, polyfluorinated alkyl substances and hepatotoxic phycotoxins are included in the Finnish monitoring program (Table 1), since 2014, to comply with the national implementation of the MSFD (e.g., Rantajärvi et al., 2021). In addition to the two publications cited in the current review, numerous publications report nodularin-R concentrations (e.g., Sivonen et al. 1989) and sedimentation in the water column (e.g., Kankaanpää et al. 2009). In addition to nodularin-R, e.g., dissolved microcystin-LR (Karlsson et al. 2005), plus saxitoxin and gonyautoxins (Hakanen et al. 2011) have been reported. The chemical analytical methods should be supported by ELISA, as currently operatively used for many phycotoxins.

**Table 1:** Monitoring activities, and targets for contaminants and their effects in pelagic Baltic Sea in Finland in 2014–2026, the latter based on Pitkänen and coworkers (2020) and Rantajärvi and co-workers (2021)

	Monitoring started (year)	Planned operativity (year)
*Contaminants*		
DDT and metabolites^b^	1985	
OTs^b,c^	2017	
PAH^c^	2017	
PBDE^b,c^	2014	
PCB^b,c^	1985	
PCDD/F^b,c^	2014	
PFAS^b,c^	2014	
Pharmaceuticals^a^	2019	
Phycotoxins (NOD and MCs)^a,b,d^	2014	
Total oil^a^	1977	
Trace elements^b,c^	1985	
*Effect determination*		
LMS^b^	2014	
*Novel approaches*		
Benthic lander, Ferrybox and research station support		2026
Drone support to oil detection		2026
Integrated chemical-biological monitoring (coastal)		2026
Oil sensors in use		2026
Passive samplers in use		2026
Satellite support to oil detection		2026

^a^water, ^b^herring, ^c^sediment, ^d^plankton

The virtual absence of EBM in Baltic Sea monitoring programs has been acknowledged for a long time. However, some national monitoring programs contain effect-based indicators such as malformations in amphipod embryos (reproductive disorders) and various physiological and health status parameters measured in fish (Sandström et al. 2005; Sundelin et al. 2018). Efforts to improve their implementation have taken place in large international projects such as the EU 5FWP project BEEP (2001**–**2004) and Baltic Sea BONUS + Programme project BEAST (2009–2011). Over 30 publications originating from these two projects only (e.g., Lehtonen and Schiedek 2006), have been published on the application of EBM in the Baltic Sea, supporting related implementation of the integrated chemical-biological monitoring. Syntheses of project results and detailed proposals for monitoring are available (Lehtonen et al. 2006, 2014). Developments from these projects have been channelled to HELCOM activities (e.g., the CORESET I and II), contributing to the listing of requirements for actions in the Baltic Sea Action Plan (2007; updated in 2021) on the development and implementation of EBM.

Apart from the above, numerous other projects and activities in the Baltic Sea have contributed to the application of EBM in monitoring. Thus, it is surprising—and worrying—that in the present review there is no mentioning of these accomplishments and outcomes of major international collaborations in this field. Although lagging a bit behind the developments achieved in, e.g., Northeast Atlantic and Mediterranean Sea, biological effects monitoring in the Baltic Sea is not starting from scratch and many field-tested methodologies are ready to be implemented. What really is lacking is the consensus in their application, and this will hopefully change soon.

None of the current EBM are specific in monitoring the impacts of this extensively heterogeneous group of chemicals. However, the great majority of them can potentially detect exposure to CEC and can be verified by subsequent chemical analyses on environmental matrices. In addition, in situ and remote sensing technologies are increasingly used in monitoring of dissolved contaminants but come with challenges of sufficient selectivity and sensitivity (overcast and attenuation in water). For example, low contaminant concentrations and optical interferences lead to detection bias, e.g., when monitoring oil-related compounds (Pärt et al. 2021).
